# Efficacy and safety of definitive chemoradiotherapy with or without induction immune checkpoint inhibitors in patients with stage III non-small cell lung cancer

**DOI:** 10.3389/fimmu.2023.1281888

**Published:** 2023-11-24

**Authors:** Yin Yang, Jianyang Wang, Tao Zhang, Zongmei Zhou, Yu Wang, Ying Jiang, Wenyang Liu, Zefen Xiao, Lei Deng, Qinfu Feng, Xin Wang, Jima Lv, Wenqing Wang, Qi Xue, Jie Wang, Ye-Xiong Li, Nan Bi

**Affiliations:** ^1^ Department of Radiation Oncology, National Cancer Center/National Clinical Research Center for Cancer/Cancer Hospital, Chinese Academy of Medical Sciences and Peking Union Medical College, Beijing, China; ^2^ Department of Thoracic Surgery, National Cancer Center/National Clinical Research Center for Cancer/Cancer Hospital, Chinese Academy of Medical Sciences and Peking Union Medical College, Beijing, China; ^3^ State Key Laboratory of Molecular Oncology, Department of Medical Oncology, National Cancer Center/National Clinical Research Center for Cancer/Cancer Hospital, Chinese Academy of Medical Sciences and Peking Union Medical College, Beijing, China; ^4^ State Key Laboratory of Molecular Oncology, Department of Radiation Oncology, National Cancer Center/National Clinical Research Center for Cancer/Cancer Hospital, Chinese Academy of Medical Sciences and Peking Union Medical College, Beijing, China

**Keywords:** stage III non-small cell lung cancer, chemoradiotherapy, immune checkpoint inhibitors, efficacy, safety

## Abstract

**Background:**

In the era of immunotherapy, the optimal combination of immune checkpoint inhibitors (ICIs) and chemoradiotherapy (CRT) for stage III non-small cell lung cancer (NSCLC) is not defined. The current study investigated the efficacy and safety of definitive CRT(dCRT) plus consolidation ICIs with or without induction ICIs in stage III NSCLC.

**Methods:**

123 consecutive patients treated with dCRT followed by consolidation ICIs at our institution from 2018 to 2022 were retrospectively reviewed. Failure patterns, survival outcomes, and toxicity profiles were analyzed.

**Results:**

The 1- and 2- year PFS rates were 75.3% and 56.9%, respectively, and median PFS was 30.83 months from the start of treatment. In-field failure (18.7%) was the most common failure pattern. The most common adverse event (AE) was pneumonitis caused by ICIs or RT. The incidence of Grade 3-4 and Grade 5 pneumonitis was 5.7% and 1.6%, respectively. Further analysis showed that the induction plus consolidation ICIs group has significantly lower cumulative incidence of distant metastasis rates (HR: 0.30, 95%CI: 0.09-1.00, p=0.043) and higher incidence of pneumonitis (p=0.039) compared with patients in the consolidation ICIs group.

**Conclusions:**

Combined CRT and consolidation ICIs achieved encouraging efficacy and manageable toxicity in patients with stage III NSCLC in China. Induction plus consolidation ICIs might reduce distant metastasis and deserve further investigation.

## Introduction

Approximately one third of patients with non–small-cell lung cancer (NSCLC) are initially diagnosed at stage III, locally advanced disease (1, 2). The results of the PACIFIC trial demonstrated significantly improved survival benefit by adding immune checkpoint inhibitors (ICIs) to definitive chemoradiotherapy (dCRT) (3). However, the updated survival analyses showed that the 5-year progression-free survival (PFS) rate was 33.1% with consolidation durvalumab, which indicated that nearly 70% of stage III NSCLC is not controlled (4). Thus, more effective treatment is urgently needed and being explored (5–8).

Novel treatment strategies used in a variety of ongoing trials are under evaluation to improve outcomes in this setting, including different anti-programmed cell death protein 1/programmed death-ligand 1(anti-PD-1/anti-PD-L1) antibodies after CRT, induction ICIs with or without chemotherapy before CRT, etc (9). The main reason for upfront ICIs before CRT is to engage immunotherapy on the basis of a healthy immune system, not impaired by chemoradiotherapy (10). Both preclinical (11) and clinical (12) studies have demonstrated that induction ICIs play a key role in activating immune surveillance against micrometastatic disease and deducing distant metastasis. Our retrospective study of induction ICIs plus chemotherapy followed by dCRT for bulky locally advanced NSCLC has shown the similar PFS and overall survival (OS) compared with those reported in the PACIFIC trial (13). Two prospective studies investigating induction ICIs followed by CRT and consolidation ICIs in stage III NSCLC, also showed the comparable PFS with PACIFIC trial (7) (14). However, cross-trial comparison is particularly challenging in these settings due to the different time of ICIs delivery and different trial inclusion and exclusion criteria.

Therefore, we assessed the benefit of the adding induction ICIs plus chemotherapy before dCRT in stage III NSCLC and exploring the optimal combination of ICIs and CRT.

## Methods

### Patient population

This study was approved by our institutional review board of Cancer Hospital, Chinese Academy of Medical Sciences. Consecutive patients treated with dCRT followed by consolidation ICIs with or without induction ICIs plus chemotherapy at our institution from September 2018 to June 2022 now were retrospectively reviewed. The inclusion criteria for the study were patients who had a pathologically confirmed unresectable stage III NSCLC according to the 8^th^ AJCC staging system, undergone dCRT and consolidation anti-PD-1/anti-PD-L1 ICIs, ≥18 years of age, >6 months follow-up. The exclusion criteria were patients who received surgery, or palliative treatment, non-completion of radiotherapy, with incomplete clinical information and so on.

### Treatment strategy

All patients received intensity modulated radiation therapy or volumetric modulated arc radiation therapy with the prescribed dose of 60 Gy, concurrently or sequentially combined with platinum-based doublet chemotherapy. Radiation therapy simulation was performed using 4-dimensional computed tomography (4D-CT) scans for all patients. The gross target volume (GTV) of the primary tumor (GTVp) was defined as the primary tumor delineated on simulation CT images, and the GTV of the lymph nodes (GTVn) was defined as any regionally involved lymph nodes with short axis >1 cm on pretreatment CT or high fluorodeoxyglucose uptake on positron emission tomography scans. For patients received induction chemotherapy with or without immunotherapy, GTVp and GTVn included the post-induction volume on the 4D-CT. The clinical target volume (CTV) comprised a margin of 0.5 cm beyond the GTV (GTVp plus GTVn) and the pre-treatment involved hilar and mediastinal nodal regions, even when the enlarged lymph nodes disappeared after induction therapy. The PTV included a margin of 0.5 cm beyond the CTV. Details of simulation, target volume definition, prescription, planning were published previously (13, 15). Patients without severe adverse events (AEs) during induction treatment and CRT could further be treated with consolidation immunotherapy for up to 1 year.

### Data collection

Baseline demographic and therapeutic data, including age, ECOG status, sex, smoking history, NSCLC pathology, cancer staging, epidermal growth factor receptor (EGFR) and PD-L1 expression, radiotherapy dose and technology, ICIs cycle and sequence, and so on, were extracted from electronic medical records. The toxicities were graded according to the Common Terminology Criteria for Adverse Events, version 5.0. During the follow-up, radiographic imaging was performed every 12 weeks for 2 years, every 6 months to 5 years, and then every year until tumor progression. The tumor response evaluation was according to Response Evaluation Criteria in Solid Tumors version 1.1. Locoregional failure (LR) was defined as clinical and/or biopsy-proven recurrence in the primary tumor or the ipsilateral hilum, mediastinum, or supraclavicular, irrespective of distant metastasis. Distant metastasis (DM) was defined as any evidence of metastatic disease beyond locoregional regions previously mentioned. Locoregional failure was further classified as in-field failure or out-field failure based on the component of disease progression occurring within or without the 95% isodose line, respectively.

### Statistical analysis

All data are expressed as median (interquartile, IQR) for continuous variables and as numbers (percentages) for categorical variables. Intergroup comparisons were performed using Mann-Whitney U test for continuous variables and the chi-square test or Fisher’s exact test when appropriate for categorical variables. One to one propensity score matching (PSM) was conducted to adjust for prespecified baseline characteristics that were potentially confounding variables. PFS was defined as the time from first treatment to the first documented event of tumor progression or death in the absence of disease progression. OS was defined as the time from first treatment to death from any cause. In the exploratory analysis for subgroup patients received consolidation ICIs, OS and PFS was further calculated from 6 weeks after RT, to better compare with the PACIFIC trial. Kaplan-Meier survival analysis was used to evaluate the PFS and OS. To properly evaluate the patterns of failure, the first site of recurrence (locoregional or distant) was analyzed by considering death as a competing risk, respectively. Univariate Cox regression model was used to calculate hazard ratio (HR) and 95% confidence interval (CI). All tests were two sided, and statistical significance was set at a p-value <0.05. Statistical analysis was performed with SPSS (version 26.0), data visualization was performed using R software (version 4.1.1).

## Result

### Patient characteristics

A total of 123 patients were identified. The baseline demographic and therapeutic characteristics of patients are presented in **Table 1**. The median age was 65 years (IQR, 57-67 years). There were 106 males (86.2%), 93 smokers (75.6%), 73 patients (59.3%) with squamous cell carcinoma, 63 patients (51.2%) presented with stage IIIB and 19 (15.4%) with stage IIIC, 79 (64.2%) patients received concurrent CRT. The median number of ICIs cycles was 12 (IQR, 6-22). 71 patients (57.7%) received the first dose of ICIs within 42 days after RT, and 67 patients (54.5%) received anti-PD-L1 ICIs which included durvalumab and sugemalimab, and other consolidation ICI drugs included pembrolizumab, camrelizumab, tislelizumab, toripalimab, sintilimab, nivolumab, atezolizumab and penpulimab. 41 patients received induction ICIs plus chemotherapy before CRT (induction plus consolidation ICIs group), all of the induction ICIs were anti-PD-1 drugs and the median number of induction ICIs cycles was 3 (IQR, 2-4). 82 patients received the first dose of ICIs followed CRT (consolidation ICIs group). Baseline characteristics were well balanced between the two groups, except a high proportion of patients in consolidated ICIs group received concurrent CRT, anti-PD-L1 ICIs, had stage N3 disease and were younger compared with induction plus consolidation ICIs group.

**Table 1:** Baseline characteristics (N=123).

	Total (n=123)	Induction plus consolidation ICIs (n=41)	Consolidation ICIs (n=82)	p
Age, median (IQR), y	63 (57-67)	66 (58.0-69.0)	62 (55.75-67.0)	0.020^*^
ECOG				0.564
0	24 (19.5)	10 (24.4)	14 (17.1)	
1	97 (78.9)	30 (73.2)	67 (81.7)	
2	2 (1.6)	1 (2.4)	1 (1.2)	
Sex				0.854
Male	106 (86.2)	35 (85.4)	71 (86.6)	
Female	17 (13.8)	6 14.6)	11 (13.4)	
Smoking history				0.373
Yes	93 (75.6)	29 (70.7)	64 (78.0)	
No	30 (24.4)	12 (29.3)	18 (22.0)	
Pathology, n(%)				0.069
Squamous cell carcinoma	73 (59.3)	29 (70.7)	44 (53.7)	
Non-squamous carcinoma	50 (40.7)	12 (29.3)	38 (46.3)	
T stage, n(%)				0.204
T1	15 (12.2)	2 (4.9)	13 (15.9)	
T2	39 (31.7)	14 (34.1)	25 (30.5)	
T3	32 (26)	14 (34.1)	18 (22)	
T4	37 (30.1)	11 (26.8)	26 (31.7)	
N stage, n(%)				0.016^*^
N0	4 (3.3)	1 (2.4)	3 (3.7)	
N1	11 (8.9)	5 (12.2)	6 (7.3)	
N2	68 (55.3)	29 (70.7)	39 (47.6)	
N3	40 (32.5)	6 (14.6)	34 (41.5)	
TNM stage, n(%)				0.211
IIIA	41 (33.3)	15 (36.6)	26 (31.7)	
IIIB	63 (51.2)	23 (56.1)	40 (48.8)	
IIIC	19 (15.4)	3 (7.3)	16 (19.5)	
PD-L1 status, n(%)				0.554
<1%	17 (13.8)	6 (14.6)	11 (13.4)	
≥1%	35 (28.5)	14 (34.1)	21 (25.6)	
NA	71 (57.7)	21 (51.2)	50 (61.0)	
EGFR status, n(%)				0.392
Mutated	4 (3.3)	1 (2.4)	3 (3.7)	
Wild type	26 (21.1)	6 (14.6)	20 (24.4)	
NA	93 (75.6)	34 (82.9)	59 (72.0)	
Concurrent CRT, n(%)				0.011^*^
Yes	79 (64.2)	20 (48.8)	59 (69.5)	
No	44 (35.8)	21 (51.2)	23 (30.5)	
ICIs cycle, median (IQR)	12 (6-22)	12 (7-21.5)	12 (5-22)	0.473
Time of ICIs post RT, n(%)				0.302
≤42	71 (57.7)	21 (51.2)	50 (61.0)	
>42	52 (42.3)	20 (48.8)	32 (39.0)	
Consolidation ICIs regimen, n(%)				<0.001^*^
Anti-PD-L1	67 (54.5)	13 (31.7)	54 (65.9)	
Anti-PD-1	56 (45.5)	28 (68.3)	28 (34.1)	

^*^p < 0.05.

IQR, interquartile range; ECOG, Eastern Cooperative Oncology Group; PD-L1, programmed death ligand-1; EGFR, epidermal growth factor receptor; CRT, chemoradiotherapy; ICIs, immune checkpoint inhibitors; RT, radiotherapy; PD-1, programmed cell death-1.

### Survival outcome

**Figure 1:**
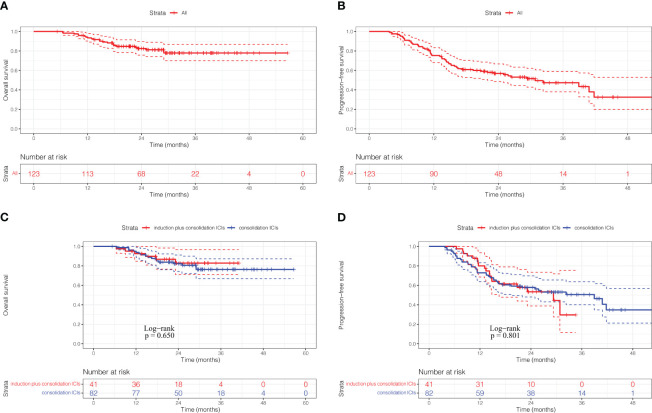
Kaplan-Meier curves of overall survival **(A)** and progression-free survival **(B)** for the full analysis set. Kaplan-Meier curves of overall survival **(C)** and progression-free survival **(D)** according to ICIs sequence. ICIs, immune checkpoint inhibitors.

With a median follow-up of 25.57 months (IQR 18.8-32.4), 52 patients (42.3%) had experienced disease progression and 23 patients (18.7%) had died. The 1-, 2- and 3- year OS rates were 94.2%, 82.5% and 77.9%, respectively, and median OS was not reached (**Figure 1A**). The 1-, 2- and 3- year PFS rates were 75.3%, 56.9% and 47.4%, respectively, and median PFS was 30.83 months for the entire cohort (**Figure 1B**). There was no significant difference in OS rate (HR: 0.81, 95%CI: 0.32-2.05, p=0.650) and PFS rate (HR: 1.07, 95%CI: 0.62-1.87, p=0.801) between the induction plus consolidation ICIs group and consolidation ICIs group (**Figures 1C, D**). However, a trend for PFS benefit with induction ICIs plus chemotherapy was observed in some subgroups, including patients with age younger than 65 years, stage N3 disease, concurrent CRT, and the number of induction ICIs cycles less than or equal to 3 (**Supplementary Figure 1**).

82 patients received the first dose of anti-PD-1/PD-L1 ICIs followed CRT, and the number of patients received durvalumab is 53, which represent the real-world data of Pacific regimen in China. The survival analysis for the 82 patients showed that the 1-, 2- and 3-year OS rates calculated from 6 weeks after RT were 90.1%, 78.9% and 76.8%, respectively, and median OS was not reached (**Supplementary Figure 2A**). The 1-, 2- and 3-year PFS rates calculated from 6 weeks after RT were 63.0%, 52.9% and 46.6%, respectively, and median PFS was 33.23 months (**Supplementary Figure 2B**).

### Duration of consolidation ICIs discontinuation

By the time of the analysis, 51(42.3%) patients have completed 12 months or more consolidation ICIs, 5 patients were continuing the consolidation ICIs, and 67 patients had discontinued consolidation ICIs due to various reasons. The most frequent cause leading to discontinuation of consolidation ICIs was pneumonitis caused by ICIs or RT, which accounted for 22.8% of the total cases, the other reasons included disease progression (19.5%), other ICIs related AEs (2.4%), pneumonia (2.4%) and patient decision (6.5%) (**Supplementary Table 1**).

### Pattern of progression

**Figure 2:**
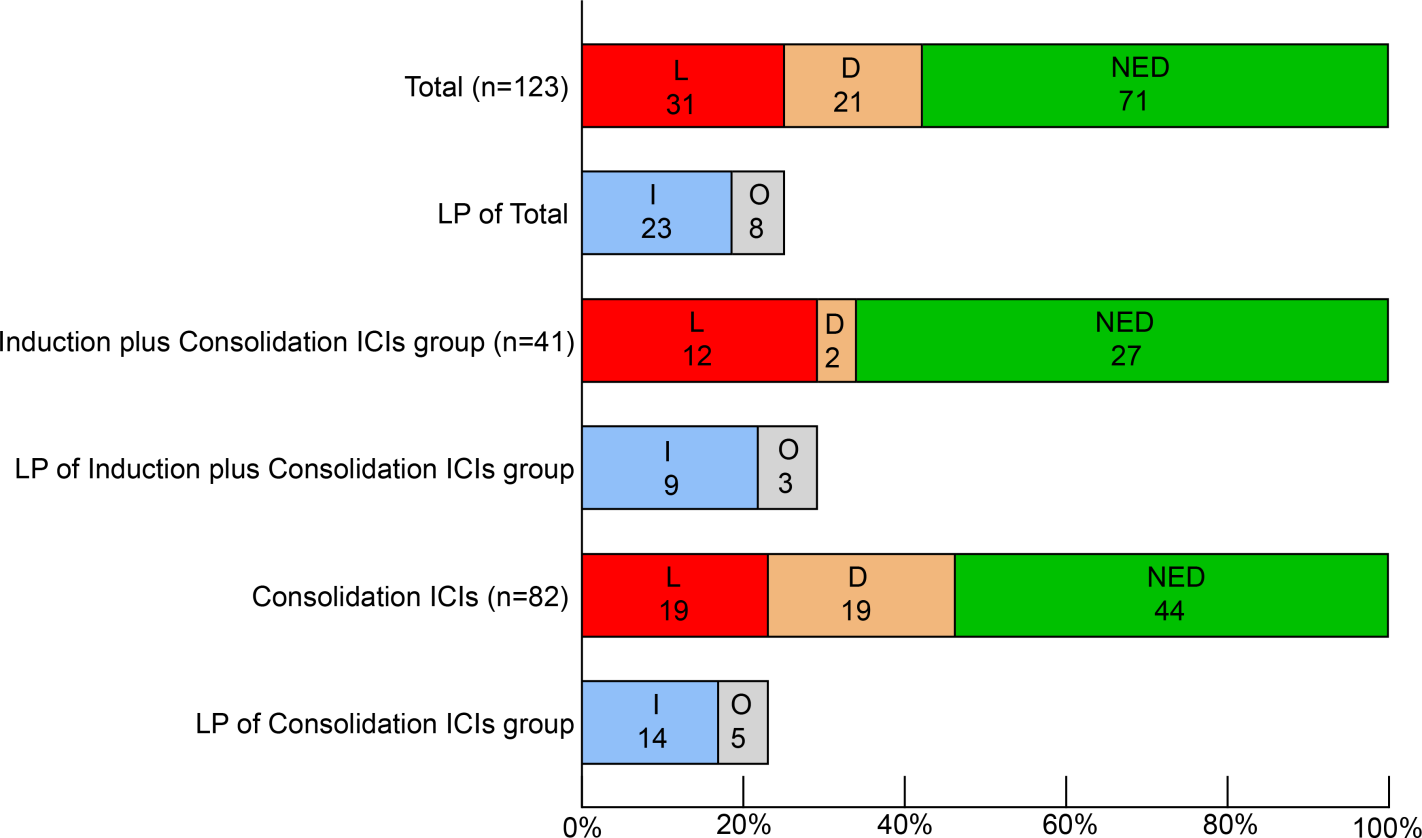
Patterns of first failure. L, locoregional progression. D, distant metastasis, NED, no evidence of disease. I, in-field failure. O, out-field failure. ICIs, immune checkpoint inhibitors.

**Figure 3:**
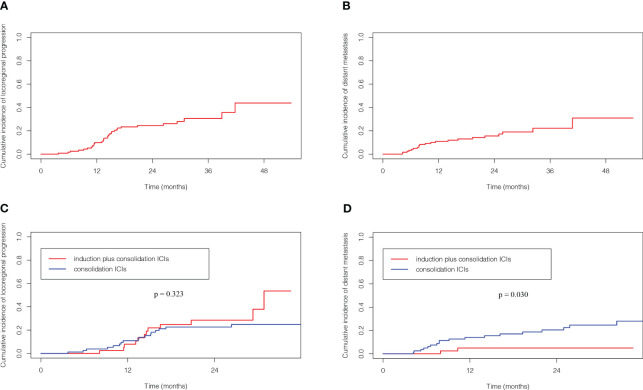
Competing risk analysis of cumulative incidence of locoregional progression **(A)** and distant metastasis **(B)** for the full analysis set. Cumulative incidence of locoregional progression **(C)** and distant metastasis **(D)** according to ICIs sequence. ICIs, immune checkpoint inhibitors.

31 patients (25.2%) experienced locoregional progression as the site of first failure, and 21 patients (17.1%) experienced distant metastasis as the site of first failure in the entire cohort (**Figure 2**). The 1-, 2- and 3- year cumulative incidence of locoregional progression rates were 9.9%, 24.5% and 30.6%, respectively (**Figure 3A**). The 1-, 2- and 3- year cumulative incidence of distant metastasis rates were 10.9%, 15.6% and 22.2%, respectively (**Figure 3B**).

The patterns of first failure were different between these two groups. Specifically, locoregional progression and distant metastasis were observed in 12 (29.3%) and 2 (4.9%) patients, respectively in the induction plus consolidation ICIs group and 19 (23.2%) and 19 (23.2%) patients, respectively in the consolidation ICIs group. And the rate of distant metastasis was significantly lower in the induction plus consolidation ICIs group than consolidation ICIs group (p=0.011) (**Supplementary Table 2**). Further analysis for the 31 patients who had locoregional progression as the site of first failure showed that in-field failure and out-field failure developed in 9 (22.0%) and 3 (7.3%) patients, respectively in the induction plus consolidation ICIs group and 14 (17.1%) and 5 (6.1%) patients, respectively in the consolidation ICIs group (**Figure 2**). There was no significant difference in cumulative incidence of locoregional progression rate (HR: 1.39, 95%CI: 0.67-2.87, p=0.323) between the induction plus consolidation ICIs group and consolidation ICIs group (**Figure 3C**). However, induction plus consolidation ICIs reduced cumulative incidence of distant metastasis rates significantly (HR: 0.23, 95%CI: 0.05-0.94, p=0.030). Specifically, the 1- and 2 - year cumulative incidence of distant metastasis rates were 4.9% and 4.9%, respectively in the induction plus consolidation ICIs group and 14.0% and 20.5%, respectively in the consolidation ICIs group (**Figure 3D**).

The incidence of new lesions is listed in **Supplementary Table 3**, the common sites were lung, lymph node, and bone, which accounts for 12.2%, 5.7% and 4.9% of the total cases, respectively. Moreover, the incidence of brain metastasis was 2.4% in this study.

### Safety profile

All adverse events in these patients are summarized in **Table 2**. In total, the incidence of Grade 1-2, Grade 3-4 and Grade 5 AEs was 96.7%, 21.1% and 1.6%, respectively. These AEs rates were similar between induction plus consolidation ICIs group and consolidation ICIs group, except significantly higher incidence of ≥Grade 3 pneumonitis was observed in induction plus consolidation ICIs group than consolidation ICIs group. Specifically, the incidence of Grade 3-4 and Grade 5 pneumonitis was 9.8% and 4.9%, respectively in the induction plus consolidation ICIs group and 3.7% and 0, respectively in the consolidation ICIs group (**Table 2**).

**Table 2:** Treatment-related adverse events.

	Total (n=123)			Induction plus consolidation ICIs (n=41)			Consolidation ICIs (n=82)			p
	Grade 1-2	Grade 3-4	Grade 5	Grade 1-2	Grade 3-4	Grade 5	Grade 1-2	Grade 3-4	Grade 5	
Any event	119 (96.7)	26 (21.1)	2 (1.6)	39 (95.1)	9 (22.0)	2 (4.9)	80 (97.6)	17 (20.7)	0	0.153
Pneumonitis^†^	81 (65.9)^#^	7 (5.7)	2 (1.6)	28 (68.3)	4 (9.8)	2 (4.9)	53 (64.6)	3 (3.7)	0	0.039^*^
Leukopenia	36 (29.3)	14 (11.4)	0	10 (24.4)	4 (9.8)	0	26 (31.7)	10 (12.2)	0	0.582
Anemia	38 (30.9)	3 (2.4)	0	14 (34.1)	0	0	24 (29.3)	3 (3.7)	0	0.266
Thrombocytopenia	21 (17.1)	6 (4.9)	0	6 (14.6)	1 (2.4)	0	15 (18.3)	5 (6.1)	0	0.535
Esophagitis	62 (50.4)	1 (0.8)	0	16 (39)	0	0	46 (56.1)	1 (1.2)	0	0.118
Radiation dermatitis	22 (17.9)	0	0	5 (12.2)	0	0	17 (20.7)	0	0	0.244
Elevated blood glucose	16 (13)	0	0	5 (12.2)	0	0	11 (13.4)	0	0	0.850
Hypothyroidism	14 (11.4)	0	0	4 (9.8)	0	0	10 (12.2)	0	0	0.526
Rash	13 (10.6)	0	0	5 (12.2)	0	0	8 (9.8)	0	0	0.681
Pruritus	13 (10.6)	0	0	3 (7.3)	0	0	10 (12.2)	0	0	0.394
Renal toxicity	13 (10.6)	0	0	3 (7.3)	0	0	10 (12.2)	0	0	0.394
Hyperthyroidism	12 (9.8)	0	0	5 (12.2)	0	0	7 (8.5)	0	0	0.685
Fatigue	6 (4.9)	0	0	2 (4.9)	0	0	4 (4.9)	0	0	1.000
Pancreatitis	6 (4.9)	3 (2.4)	0	3 (7.3)	2 (4.9)	0	3 (3.7)	1 (1.2)	0	0.324
Musculoskeletal pain	5 (4.1)	0	0	0 (0)	0	0	5 (6.1)	0	0	0.168
Myocarditis	3 (2.4)	0	0	1 (2.4)	0	0	2 (2.4)	0	0	1.000
Vascular hyperplasia	2 (1.6)	0	0	2 (4.9)	0	0	0 (0)	0	0	0.109
Cough	2 (1.6)	0	0	0 (0)	0	0	2 (2.4)	0	0	0.552
Peripheral neuropathy	1 (0.8)	0	0	0 (0)	0	0	1 (1.2)	0	0	1.000

^*^p < 0.05.

^†^including radiation pneumonitis and immune-related pneumonitis.

^#^With Grade 1 being 26.0% and Grade 2 being 39.8%.

ICIs, immune checkpoint inhibitors.

### PSM analysis for induction plus consolidation group versus consolidation group

After adjusting for confounding variables via PSM, all clinical features were balanced between the induction plus consolidation ICIs group and consolidation ICIs group (**Supplementary Table 4**). The OS rate (HR: 0.77, 95%CI: 0.27-2.17, p=0.619), PFS rate (HR: 0.99, 95%CI: 0.53-1.84, p=0.963) and cumulative incidence of locoregional progression rate (HR: 0.97, 95%CI: 0.44-2.16, p=0.979) were similar between the induction plus consolidation ICIs group and consolidation ICIs group (**Supplementary Figures 3A–C**). However, patients in the induction plus consolidation ICIs group had significantly lower cumulative incidence of distant metastasis rates compared with patients in the consolidation ICIs group (HR: 0.21, 95%CI: 0.05-0.92, p=0.032). Specifically, the 1- and 2- year cumulative incidence of distant metastasis rates were 4.9% and 4.9%, respectively in the induction plus consolidation ICIs group, and 16.1% and 19.8%, respectively in the consolidation ICIs group (**Supplementary Figure 3D**). As for the toxicity, except significantly higher incidence of ≥Grade 3 pneumonitis was observed in induction plus consolidation ICIs group than consolidation ICIs group (p=0.049), these rates of other AEs were similar for patients in both groups (**Supplementary Table 5**).

## Discussion

This is the first study to compare the dCRT followed by consolidation ICIs with or without induction ICIs plus chemotherapy in one cohort of unresectable, stage III NSCLC patients. This study identified encouraging efficacy and manageable toxicity of the combined ICIs and CRT for patients with stage III NSCLC, and demonstrated that induction and consolidation ICIs might reduce distant metastasis, while achieve similar survival benefit compared with consolidation ICIs.

Since the publication of the PACIFIC trial (3) in 2017 which demonstrated significantly improved survival benefit under the addition of consolidation ICIs followed by CRT in unresectable, stage III NSCLC patients, other novel treatment strategies including induction ICIs before CRT given to engage with a healthy immune system have been used in a variety of ongoing trials (7, 14). In this retrospective study, all the patients received CRT and consolidation ICIs, the current standard of care, among whom some patients received induction ICIs plus chemotherapy because of patient’s willingness to undergo surgery or challenging to initial definitive CRT. The survival analysis showed similar OS, PFS and cumulative incidence of locoregional progression between the two treatment groups. Notably, the induction plus consolidation ICIs group achieved significantly lower distant metastasis rate compare with consolidation ICIs group, which is reasonable as both preclinical (11) and clinical (12) studies have demonstrated that induction ICIs play a key role in activating immune surveillance against micrometastatic disease and reducing distant metastasis. However, the reduced distant metastasis was not translated into a PFS or OS benefit in the induction ICIs group. Although the exact reasons are not known, we think it could be explained by the following aspects. First, given that the rate of distant metastasis has already been significantly reduced for patients with stage III NSCLC under the treatment of consolidation ICIs (4), the further reduced distant metastasis by the adding induction ICIs plus chemotherapy exerted limited effects on improving PFS or OS, especially in this cohort whose dominant pattern of failure was locoregional progression. Second, a significantly higher incidence of ≥Grade 3 pneumonitis in the induction plus consolidation ICIs group was observed in this cohort, which exerted negative impact on PFS and OS (16). Based on these results, we get the preliminary conclusion that different ICIs strategies were needed based on the tumor characteristic and the potential failure pattern. In our retrospective study for bulky tumor, which was defined as primary tumor ≥5 cm in greatest dimension or regionally involved lymph nodes ≥2 cm in shortest diameter, has shown that induction ICIs plus chemotherapy played a key role in shrinking tumor volume and achieved the similar prognosis compared with PACIFIC trial (13). However, excessive induction ICIs plus chemotherapy may result in early pneumonitis or tumor progression, which delaying or discontinuing the following definitive CRT and consolidation ICIs (13). Last, the finding should be interpreted with caution given the retrospective nature, moderate sample size, and heterogeneous ICI agents of our study. Nevertheless, this study provides preliminary evidence, and well-designed randomized studies with large sample sizes are warranted to validate these conclusions. Moreover, individualized therapy according to other tumor characteristic and biomarker is lacking and deserve further investigation.

By the time of the analysis, about half of these patients (42.3%) completed 12 months or more consolidation ICIs in our cohort, which is close to those reported in Pacific-R trial (47.1%) and Pacific trial (48.7%) (17). The incidence of new lesions was 23.6%, and the most frequent sites were lung and lymph node in this study, which was consistent with those reported in PACIFIC trial (4). In addition, locoregional progression was the dominant pattern of first failure in our cohort. Analysis for the patients who had locoregional progression showed that in-field failure(18.7%) was more common compared with out-field failure(6.5%). Noriko et al. (18) also reported that in-field recurrence was the most common locoregional progression pattern in patients with stage III NSCLC received CRT followed ICIs, which is consistent with our study. Further improvement of the in-field control is still a major problem even in the ICIs era.

The survival rates in this study were numerically superior to those in the PACIFIC trial (4) and Pacific-R trial (19). In the PACIFIC trial (4), the 1-, 2- and 3-year OS rates were 83.1%, 66.3% and 56.7%, respectively. The 1-, 2- and 3-year PFS rates were 55.7%, 45.0% and 39.7%, respectively, and the median PFS was 16.9 months. And in Pacific-R trial (19), the 2-year OS and PFS rates were 71.2% and 48.2%, respectively. Two possible reasons exist for the superior treatment effect of our results with previous reports. First, Asian patients with NSCLC under the treatment of ICIs gain better prognosis compared with non-Asian patients. As the multivariable cox regression analysis in the PACIFIC trial showed that Asian, accounted for 26.9% of the total cases, is independent protective factor of OS (4). In addition, the real-world data for Korean from PACIFIC-KR trial also showed superior PFS and OS than those reported in PACIFIC trial (4) and Pacific-R trial (19). Second, assessments after treatment could sometimes be delayed in retrospective study, which led to overestimated prognosis.

In terms of toxicity, the most common AEs was pneumonitis caused by ICIs or RT. In some cases, the differentiation between radiation pneumonitis and immune-relate pneumonitis was difficult. The overall pneumonitis incidence was markedly higher (65.9%) than it reported in the PACIFIC trial (33.9%) (3). Two possible reasons exist for the discrepancy of our results with previous reports in the incidence of pneumonitis. First, the incidence of pneumonitis varies by race/ethnicity. A meta-analysis of real-world studies has been demonstrated that the incidence of pneumonitis was about three times higher to Asian populations than non-Asian populations (20). Given that Asian population accounted for only 9% of the total cases in PACIFIC trial but 100% of our study, it is understandable that the incidence of pneumonitis is markedly higher than it reported in the PACIFIC trial. Second, the incidence of pneumonitis varies by anti-PD-1/PD-L1 ICIs. A meta-analysis had indicated that the incidence of pneumonitis with use of anti-PD-1 ICIs was about three times higher than anti-PD-L1 ICIs (21). A valid hypothesis explaining the discrepancy is that anti-PD-1 blockade may shift the balance in PD-L2 interaction with its other binding partners and lead to pneumonitis (22, 23). Considering that 56.1% of patients received anti-PD-1 ICIs in this study, it is reasonable that the incidence of pneumonitis is markedly higher. However, the treatment was overall well-tolerated and most of the pneumonitis was Grade 1-2 (with Grade 1 being 26.0% and Grade 2 being 39.8%) and clinically manageable. The incidence of Grade 3-4 and Grade 5 pneumonitis was 5.7% and 1.6%, respectively, which is similar to the patients with induction and consolidation ICIs in the KEYNOTE-799 trial (5% and 5%, respectively) (8).

There are several limitations in this study. Firstly, this is a single institution retrospective analysis. Secondly, not long follow-up time and significantly improved treatment effect in this study gave rise to limited events of tumor progression and death, a longer follow-up may be more informative. However, under the circumstance that there is no study to investigate the optimal combination of ICIs and CRT for LA-NSCLC, our analysis can provide a rationale for the design RCTs, as well as clinical practice. Future RCTs are expected to validate and update these results.

## Conclusion

This is the first study to evaluate the dCRT followed by consolidation ICIs with or without induction ICIs plus chemotherapy in one cohort of stage III NSCLC patients. This study identified encouraging efficacy and manageable toxicity of the combined ICIs and CRT for patients with stage III NSCLC in China, and demonstrated that induction plus consolidation ICIs could reduce distant metastasis and gain similar survival benefit compared with consolidation ICIs.

## Data availability statement

The raw data supporting the conclusions of this article will be made available by the authors, without undue reservation.

## Ethics statement

The studies involving humans were approved by National Cancer Center/National Clinical Research Center for Cancer/Cancer Hospital, Chinese Academy of Medical Sciences and Peking Union Medical College. The studies were conducted in accordance with the local legislation and institutional requirements. The ethics committee/institutional review board waived the requirement of written informed consent for participation from the participants or the participants’ legal guardians/next of kin because of the retrospective nature of this research.

## Author contributions

YY: Data curation, Formal analysis, Methodology, Writing – original draft. JYW: Investigation, Methodology, Resources, Writing – original draft. TZ: Data curation, Resources, Writing – review & editing. ZZ: Data curation, Resources, Writing – review & editing. YW: Data curation, Methodology, Writing – review & editing. YJ: Data curation, Methodology, Writing – review & editing. WL: Investigation, Resources, Writing – review & editing. ZX: Investigation, Resources, Writing – review & editing. LD: Investigation, Resources, Writing – review & editing. QF: Investigation, Resources, Writing – review & editing. XW: Investigation, Resources, Writing – review & editing. JL: Investigation, Resources, Writing – review & editing. WW: Investigation, Resources, Writing – review & editing. QX: Conceptualization, Resources, Writing – review & editing. JW: Conceptualization, Resources, Writing – review & editing. Y-XL: Conceptualization, Project administration, Supervision, Writing – review & editing. NB: Conceptualization, Funding acquisition, Supervision, Writing – review & editing.
