# Supplementary material for: Efficacy and safety of definitive chemoradiotherapy with or without induction immune checkpoint inhibitors in patients with stage III non-small cell lung cancer
Source: Front Immunol. 2023 Nov 24;14:1281888. doi: 10.3389/fimmu.2023.1281888 (PMC10704131; doi:10.3389/fimmu.2023.1281888)
Supplement: Supplementary file 1 [file DataSheet_1.docx]

**Supplementary tables**

**Supplementary Table 1. Reasons for consolidation ICIs discontinuation.**

|  | Patients, No.(%) |
| --- | --- |
| Completed treatment | 52 (42.3) |
| Ongoing  Discontinuation | 5 (4.1) |
| Pneumonitis | 28 (22.8) |
| Disease progression | 24 (19.5) |
| Other ICIs-related AEs | 3 (2.4) |
| Pneumonia | 3 (2.4) |
| Patient decision | 8 (6.5) |

Abbreviations: ICIs = immune checkpoint inhibitors; AEs= adverse events.

**Supplementary Table 2. Patterns of first failure**

| Location | Total (n=123) | Induction plus consolidation ICIs (n=41) | Consolidation ICIs (n=82) | p |
| --- | --- | --- | --- | --- |
| Any | 52 (42.3) | 14 (34.1) | 38 (46.3) | 0.197 |
| Distant metastasis | 21 (17.1) | 2 (4.9) | 19 (23.2) | 0.011^*^ |
| Locoregional progression | 31 (25.2) | 12 (29.3) | 19 (23.2) | 0.463 |
| In-field | 23 (18.7) | 9 (22.0) | 14 (17.1) | 0.513 |
| Out-field | 8 (6.5) | 3 (7.3) | 5 (6.1) | 0.796 |

Abbreviations: ICIs = immune checkpoint inhibitors.

^*^p < 0.05.

**Supplementary Table 3. Incidence of new lesions.**

| New Lesion Site^#^ | Patients, No.(%) |
| --- | --- |
| Any | 29 (23.6) |
| Lung | 15 (12.2) |
| Lymph nodes | 7 (5.7) |
| Bone | 6 (4.9) |
| Brain | 3 (2.4) |
| Liver | 3 (2.4) |
| Adrenal gland | 1 (0.8) |
| Kidney | 1 (0.8) |

^#^ A patient could have more than one new lesion site.

**Supplementary Table 4. Baseline characteristics among the two groups after PSM (N=82)**

|  | Induction plus consolidation ICIs (n=41) | Consolidation ICIs (n=41) | p |
| --- | --- | --- | --- |
| Age, median (IQR), y | 66 (58.0-69.0) | 63 (55.5-67.0) | 0.079 |
| ECOG |  |  |  |
| 0 | 10 (24.4) | 5 (12.2) | 0.353 |
| 1 | 30 (73.2) | 35 (85.4) |  |
| 2 | 1 (2.4) | 1 (2.4) |  |
| Sex |  |  | 0.480 |
| Male | 35 (85.4) | 38 (92.7) |  |
| Female | 6 (14.6) | 3 (7.3) |  |
| Smoking history |  |  | 0.109 |
| Yes | 29 (70.7) | 35 (85.4) |  |
| No | 12 (29.3) | 6 (14.6) |  |
| Pathology, n(%) |  |  | 0.635 |
| Squamous cell carcinoma | 29 (70.7) | 27 (65.9) |  |
| Non-squamous carcinoma | 12 (29.3) | 14 (34.1) |  |
| T stage |  |  | 0.244 |
| T1 | 2 (4.9) | 6 (14.6) |  |
| T2 | 14 (34.1) | 9 (22.0) |  |
| T3 | 14 (34.1) | 11 (26.8) |  |
| T4 | 11 (26.8) | 15 (36.6) |  |
| N stage |  |  | 0.271 |
| N0 | 1 (2.4) | 3 (7.3) |  |
| N1 | 5 (12.2) | 6 (14.6) |  |
| N2 | 29 (70.7) | 21 (51.2) |  |
| N3 | 6 (14.6) | 11 (26.8) |  |
| TNM stage, n(%) |  |  | 0.875 |
| IIIA | 15 (36.6) | 16 (39.0) |  |
| IIIB | 23 (56.1) | 21 (51.2) |  |
| IIIC | 3 (7.3) | 4 (9.8) |  |
| PD-L1 status, n(%) |  |  | 0.602 |
| ＜1% | 6 (14.6) | 6 (14.6) |  |
| ≥1% | 14 (34.1) | 10 (24.4) |  |
| NA | 21 (51.2) | 25 (61.0) |  |
| EGFR status, n(%) |  |  | 0.481 |
| Mutated | 1 (2.4) | 0 |  |
| Wild type | 6 (14.6) | 7 (17.1) |  |
| NA | 34 (82.9) | 34 (82.9) |  |
| Concurrent CRT, n(%) |  |  | 0.118 |
| Yes | 20 (48.8) | 27 (65.9) |  |
| No | 21 (51.2) | 14 (34.1) |  |
| ICIs cycle, median (IQR) | 12 (7-21.5) | 10 (4-17) | 0.206 |
| Time of ICIs post RT, n(%) |  |  | 0.373 |
| ≤42 days | 21 (51.2) | 25 (61.0) |  |
| >42 days | 20 (48.8) | 16 (39.0) |  |
| Consolidation ICIs regimen, n(%) |  |  | 0.174 |
| Anti-PD-L1 | 13 (31.7) | 22 (53.7) |  |
| Anti-PD-1 | 28 (68.3) | 19 (46.3) |  |

Abbreviations: PSM = propensity score matching; IQR = interquartile range; ECOG = Eastern Cooperative Oncology Group; PD-L1= programmed death ligand-1; EGFR = epidermal growth factor receptor; CRT= chemoradiotherapy; ICIs = immune checkpoint inhibitors; RT=radiotherapy; PD-1= programmed cell death-1.

**Supplementary Table 5. Treatment-related adverse events^#^ after PSM.**

|  | Induction plus consolidation ICIs (n=41) | | | consolidation ICIs (n=41) | | | p |
| --- | --- | --- | --- | --- | --- | --- | --- |
|  | Grade 1-2 | Grade 3-4 | Grade 5 | Grade 1-2 | Grade 3-4 | Grade 5 |  |
| Any event | 39 (95.1) | 9 (22.0) | 2 (4.9) | 40 (97.6) | 8 (19.5) | 0 | 0.421 |
| Pneumonitis^†^ | 28 (68.3) | 4 (9.8) | 2 (4.9) | 25 (61.0) | 1 (2.4) | 0 | 0.049^*^ |
| Leukopenia | 10 (24.4) | 4 (9.8) | 0 | 12 (29.3) | 5 (12.2) | 0 | 0.791 |
| Anemia | 14 (34.1) | 0 | 0 | 10 (24.4) | 3 (7.3) | 0 | 0.089 |
| Thrombocytopenia | 6 (14.6) | 1 (2.4) | 0 | 4 (9.8) | 4 (9.8) | 0 | 0.310 |
| Esophagitis | 16 (39) | 0 | 0 | 24 (58.5) | 1 (2.4) | 0 | 0.077 |

Abbreviations: PSM = propensity score matching; ICIs = immune checkpoint inhibitors.

^#^reported in ≥15% of patients.

^†^including radiation pneumonitis and immune-related pneumonitis.

^*^p < 0.05.

**Supplementary Figures**


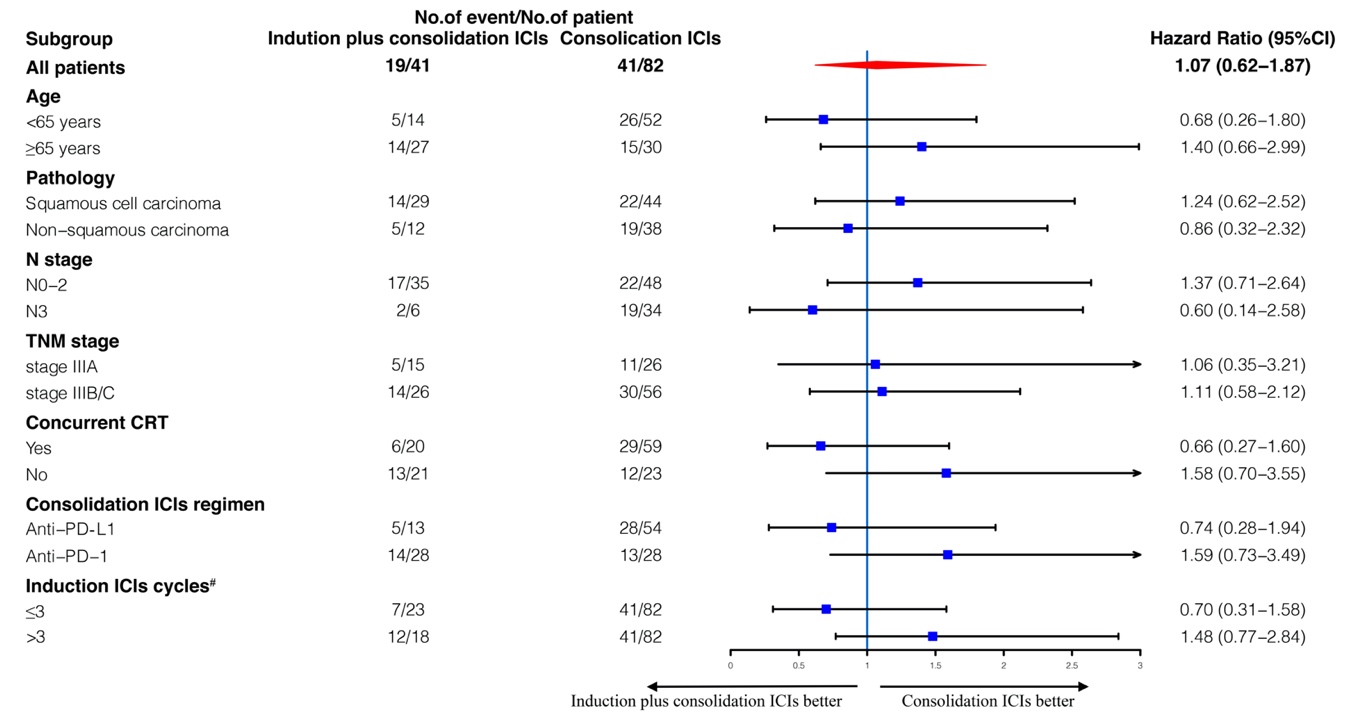


Supplementary Figure 1. Subgroup analysis of prognostic factors for progression-free survival. ^#^For the patients in induction plus consolidation ICIs group.

B

A

A

Supplementary Figure 2. Survival analysis of consolidation ICIs group. (A)Overall survival. (B)Progression-free survival. ICIs, immune checkpoint inhibitors.

D

C

B

A

Supplementary Figure 3. Kaplan-Meier curves of overall survival(A), progression-free survival(B), cumulative incidence of locoregional progression(C) and distant metastasis(D) according to ICIs sequence after PSM. ICIs, immune checkpoint inhibitors. PSM, propensity score matching.
